# Risk factors and clinical characteristics of *Pneumocystis jirovecii* pneumonia in lung cancer

**DOI:** 10.1038/s41598-019-38618-3

**Published:** 2019-02-14

**Authors:** Eun Hye Lee, Eun Young Kim, Sang Hoon Lee, Yun Ho Roh, Ah Young Leem, Joo Han Song, Song Yee Kim, Kyung Soo Chung, Ji Ye Jung, Young Ae Kang, Young Sam Kim, Joon Chang, Moo Suk Park

**Affiliations:** 1 Division of Pulmonology, Department of Internal Medicine, Institute of Chest Diseases, Severance Hospital, Yonsei University College of Medicine, Seoul, Republic of Korea; 2 Biostatistics Collaboration Unit, Yonsei University College of Medicine, Seoul, Republic of Korea

## Abstract

Solid malignancies are associated with the development of *Pneumocystis jirovecii* pneumonia (PJP). This study aimed to evaluate the risk factors for PJP among patients with lung cancer. This retrospective case-control study compared patients who had lung cancer with PJP (n = 112) or without PJP (n = 336) matched according to age, sex, histopathology, and stage. PJP definition was based on (i) positive PCR or direct immunofluorescence results for pneumocystis, (ii) clinical symptoms and radiological abnormalities that were consistent with a pneumonic process, and (iii) received targeted PJP treatment. The development of PJP was associated with radiotherapy (RTx), concurrent chemoradiotherapy (CCRTx), lymphopenia, and prolonged high-dose steroid therapy (20 mg of prednisolone equivalent per day for ≥3 weeks). Multivariate analysis revealed independent associations with prolonged high-dose steroid therapy (odds ratio [OR]: 1.96, 95% confidence interval [CI]: 1.06–3.63; *p* = 0.032) and CCRTx (OR: 2.09, 95% CI: 1.27–3.43; *p* = 0.004). Steroid use was frequently related to RTx pneumonitis or esophagitis (29 patients, 43.3%). Prolonged high-dose steroid therapy and CCRTx were risk factors for PJP development among patients with lung cancer. As these patients had a poor prognosis, clinicians should consider PJP prophylaxis for high-risk patients with lung cancer.

## Introduction

*Pneumocystis jirovecii* pneumonia (PJP) is an opportunistic infection caused by the fungus *Pneumocystis jirovecii*^1^. During 1980–2000, most PJP cases involved patients with acquired immunodeficiency syndrome (AIDS), although PJP has become common in non-human immunodeficiency virus (HIV)-infected cases because of improvements in AIDS treatment and more patients receiving immunosuppressive therapy^2^. However, non-HIV-infected patients with PJP have an overall mortality rate of approximately 35–55%^3–5^, compared to 10–20% among patients with AIDS^6^.

*Hughes et al*.^7^ reported that the incidence of PJP in acute lymphoblastic leukemia (ALL) ranged from 22% to 45%, depending on the chemotherapy used and the stage of leukemia. Another study also reported that without prophylaxis, PJP rates may approach 25% among patients with non-Hodgkin’s lymphoma^8^. In a recent study, PJP incidence of acute leukemia, chronic lymphocytic leukemia, and non-Hodgkin lymphoma was estimated to be >45 cases per 100,000 patient year, whereas PJP incidence of solid tumors was <25 cases per 100,000 patient-year^9^. Possible explanations of higher incidence of PJP in hematologic malignancies are related to high intensity of chemotherapy^7^ and severely impaired immune function^10^. After routine performance of prophylaxis using trimethoprim-sulfamethoxazole (TMP-SMX), incidence of PJP among patients with hematologic malignancies has shown remarkable decrease^11^.

Although the exact incidence of PJP in solid tumors still remains unknown, recent reports have reported PJP cases in lung cancer, which is the most common cause of solid malignancy-related death^3,9,12^. However, little is known about the risk factors for PJP development and who would benefit from PJP prophylaxis in lung cancer patients. Therefore, this study aimed to evaluate the risk factors for PJP among patients with lung cancer.

## Patients and Methods

### Study design and population

**Figure 1:**
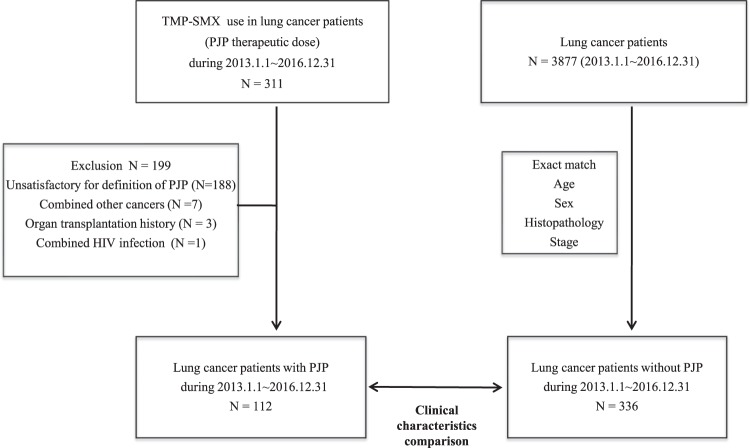
Flowchart of study patients. TMP-SMX; trimethoprim-sulfamethoxazole, PJP; pneumocystis jirovecii pneumonia, HIV; human immunodeficiency virus.

Electronic medical records from the Severance Hospital (a 2,500-bed tertiary referral hospital in South Korea) were searched to identify 3,877 patients with lung cancer who underwent surgery, chemotherapy (CTx), and/or radiotherapy (RTx) between January 2013 and December 2016. Among these patients, 311 patients had received treatment for PJP using intravenous and/or oral TMP-SMX (trimethoprim: 15–20 mg/kg/day, sulfamethoxazole: 75–100 mg/kg/day). However, cases were excluded if they had an unsatisfactory definition of PJP (n = 188), other cancers (n = 7), organ transplantation (n = 3), or HIV infection (n = 1). Thus, records from 112 patients with confirmed lung cancer and PJP were matched 1:3 with control patients (n = 336, lung cancer without PJP) according to age, sex, histopathology, and stage using exact match modeling (Fig. 1). Treatment modalities of lung cancer, including types of chemotherapy, were determined by relevant physician on a case-by-case basis, and treatment principles were based on national comprehensive cancer network (NCCN) guideline over study period^13,14^.

### PJP Definition

PJP was considered present when the patient fulfilled three conditions. The first condition was a positive result for *Pneumocystis jirovecii* using either real-time PCR testing or direct immunofluorescence testing of spontaneous sputum, induced sputum, bronchoalveolar lavage (BAL), or tissue samples. The real-time PCR assay was performed with the AmpliSens *Pneumocystis jirovecii*-FRT PCR kit (Moscow, Russia), which is a qualitative test based on the endogenous control, the β-globin gene. Ten-microliter DNA samples were added in each reaction tubes. PCRs were performed on the Bio-Rad CFX-96 real-time PCR system (Bio-Rad, Hercules, CA) according to the manufacturer’s instruction. The second condition was the presence of lung infiltration during chest radiography or computed tomography (CT)^10,15,16^. The third condition was clinical symptoms of PJP and PJP treatment during their hospitalization. We defined the prophylaxis group as those without a PJP diagnosis who received medication to prevent PJP.

### Data collection

Demographic and clinical data were collected through a chart review. The lowest total lymphocyte count was selected from the 2 weeks before the PJP diagnosis (PJP group) or during the follow-up period (control group), and lymphopenia was defined as <1,000 cells/µL^17^. The daily dosage of steroids was expressed in prednisolone equivalents, and the continuous maximum use of steroids was recorded as the cumulative steroid dose. We did not consider intermittent steroid use (e.g., as emesis prophylaxis during CTx), or steroid use that was stopped at >1 month before the PCP diagnosis, in order to more accurately account for the effect of steroid treatment on PJP development^18^. Steroid dosage during the last 2 weeks before the PJP diagnosis was compared to the dosage during the preceding weeks, and changes during the 2 last weeks were categorized as maintained, increased, tapered, or stopped^17^. Adjunctive steroid use during PJP treatment was defined a high-dose (≥60 mg/day prednisolone equivalent), low-dose (<60 mg/day prednisolone equivalent), or absent^19^. The 60-mg cut-off was selected because it was the lowest effective dose in controlled trials for HIV-infected patients with PJP^1,20^. RTx-induced pneumonitis was diagnosed based on the patients’ symptoms (low-grade fever, dry cough, dyspnea, or chest pain) and radiological manifestations^21,22^. Radiation-induced esophagitis was also defined based on clinical symptoms (dysphagia, odynophagia, and substernal discomfort)^23^. Data from chest radiography and CT were evaluated independently by one radiologist at the PJP diagnosis, and then independently confirmed by two of the authors (EH Lee, MS Park). Medical records related to risk factors for PJP development were studied until PJP development in PJP patients, whereas those in controls were studied until death, transfer to another hospital, or December 2016.

### Statistical analysis

Normally distributed continuous variables were reported as mean ± standard deviation, while non-normally distributed variables were reported as median (range). Continuous variables were analyzed using the Student t-test or the Mann-Whitney test. Categorical variables were reported as number and percentage, and compared using the chi-square test or Fisher’s exact test. The independent risk factors for PJP development were evaluated using conditional logistic regression modeling based on the matched paired data, and the results were reported as odds ratios (ORs) and 95% confidence intervals (CIs). A two-tailed P-value of <0.05 was considered statistically significant. All analyses were performed using SAS software (version 9.4; SAS Inc., Cary, NC).

### Ethics

The research protocol was approved by the Institutional Review Board (IRB) of Severance Hospital (IRB No. 4-2017-0431). The need for informed consent was waived due to the retrospective nature of the study. All methods were carried out in accordance with relevant guidelines and regulations.

## Results

### Clinical characteristics and outcomes for PJP

The most common symptom of PJP was dyspnea (71.4%), followed by fever (27.7%) and cough (23.2%) (Table 1). The median respiratory symptom duration was 3 days (range: 1–20 days). Sixty-seven patients (59.8%) received steroids within 1 month before developing PJP. The most common reasons for steroid treatment were RTx complications like RTx pneumonitis or esophagitis (29 patients, 43.3%), relief of tumor infiltration symptoms (24 patients, 35.8%) such as increased intracranial pressure, bronchus obstruction, spinal cord compression and adrenal insufficiency (8 patients, 11.9%). Six patients (8.9%) received steroids because of exacerbated underlying lung disease. At the PJP diagnoses, 33 patients (49.3%) had a maintained dosage, 23 patients (34.3%) had a tapered dosage, and 11 patients (16.4%) had stopped steroid treatment. The most common findings were diffuse interstitial infiltrates during radiography (66.9%) and bilateral ground-glass attenuation during CT (66.3%). Focal consolidation and ground-glass opacities were also detected.

**Table 1:** Clinical characteristics for PJP among patients with lung cancer.

Characteristics (N = 112)	Data
Age (years), median (range)	69 (42–88)
Sex, male, n (%)	93 (83.0)
Time from diagnosis of lung cancer (days), median (range)	197 (25–1114)
Time from respiratory symptom onset (days), median (range)	3 (1–20)
Respiratory symptom at admission, n (%)	
Dyspnea	80 (71.4)
Fever	31 (27.7)
Cough	26 (23.2)
Sputum	22 (19.6)
Others*	16 (14.3)
Patients receiving PJP prophylaxis, n (%)	3 (2.7)
Steroid use, n (%)	67 (59.8)
Reasons for steroid use, n (%)	
RTx complication (RTx pneumonitis, RTx induced esophagitis)	25 + 4/67 (43.3)
IICP control for brain metastasis	16/67 (23.9)
Adrenal insufficiency	8/67 (11.9)
Underlying ILD or COPD exacerbation	6/67 (8.9)
Bronchus obstruction due to tumor infiltrate	4/67 (6.0)
Cord compression due to spine metastasis	4/67 (6.0)
Maintained, n (%)	33/67 (49.3)
Tapered, n (%)	23/67 (34.3)
Stopped, n (%)	11/67 (16.4)
Chest x-ray findings at admission, n (%)	112 (100)
Diffuse interstitial infiltrate, n (%)	75/112 (66.9)
Unilateral (or focal) interstitial infiltrates, n (%)	26/112 (23.2)
Focal consolidation, n (%)	11/112 (9.8)
Chest CT scan findings, n (%)^†^	92/112 (82.1)
Diffuse ground glass attenuation, n (%)	61/92 (66.3)
Focal ground glass attenuation, n (%)	20/92 (21.7)
Focal consolidation, n (%)	7/92 (7.6)
Others, n (%)^§^	4/92 (4.4)

Data are presented as median (range) or numbers (percentages) unless otherwise indicated.

*Hemoptysis, chest pain, general weakness, poor oral intake.

^†^20 patients could not take the CT scan due to poor general condition and high oxygen demand.

^§^Cystic lesion, combination of ground glass attenuation and consolidation, solitary or multiple nodules, linear reticular opacities.

PJP, *pneumocystis jirovecii* pneumonia; RTx, radiotherapy; IICP, increased intracranial pressure; ILD, interstitial lung disease; COPD, chronic obstructive pulmonary disease.

Table 2 shows treatment outcomes of PJP patients with lung cancer. All patients had received TMP-SMX, although 7 patients (6.3%) required second-line medication due to progression of PJP despite TMP-SMX therapy (primaquine and clindamycin in 6 cases, pentamidine in 1 case). Seventy-five patients (67%) received high-dose adjunctive steroids during PJP treatment, 26 patients (23.2%) received low-dose adjunctive treatment, and 11 patients (9.8%) did not receive adjunctive steroids. The median duration of PJP treatment was 13 days (range: 2–89 days), and 69 of the 112 patients (61.6%) died during PJP treatment (respiratory failure: 72.5%, cancer progression: 14.5%, and respiratory failure with cancer progression: 13.0%). Among the 69 deaths, 47 deaths (68.1%) involved a do-not-resuscitate and do-not-intubate order, and only 6 patients (8.7%) had a full code status.

**Table 2:** Treatment outcomes for PJP among patients with lung cancer.

Treatment outcomes (N = 112)	Data
Treatment regimen	
TMP-SMX alone, n (%)	105/112 (93.7)
TMP-SMX prior to primaqiune + clinadamycin, n (%)	6/112 (5.4)
TMP-SMX prior to pentamidine, n (%)	1/112 (0.9)
Adjunctive steroid, n (%)	
High dose, ≥60 mg/day prednisolone equivalent	75/112 (67.0)
Low dose, <60 mg/day prednisolone equivalent	26/112 (23.2)
Not used	11/112 (9.8)
Duration of PJP treatment (days), median (range)	13 (2–89)
Death during PJP Treatment, n (%)	69/112 (61.6)
Immediate cause of death	
Respiratory failure due to PJP	50/69 (72.5)
Cancer progression	10/69 (14.5)
Respiratory failure due to PJP + cancer progression	9/69 (13.0)
Code status	
Full code	6/69 (8.7)
DNR + intubation	16/69 (23.2)
DNR including DNI	47/69 (68.1)

Data are presented as median (range) or numbers (percentages) unless otherwise indicated.

PJP, *pneumocystis jirovecii* pneumonia; TMP-SMX, trimethoprim-sulfamethoxazole; DNR, do not resuscitate; DNI, do not intubate.

### Baseline characteristics of patients according to PJP status

Table 3 shows the clinical characteristics of the matched patients with and without PJP. The median patient age was 69 years (range: 42–88), and most patients were men (83%). The most frequent histopathological types were adenocarcinoma (46.4% vs. 49.4%), squamous cell carcinoma (36.6% vs. 35.7%), and small cell lung cancer (12.5% vs. 14.3%). Approximately 75% of the patients had stage IV lung cancer. More than 90% of the patients received CTx (94.6% vs. 92.6%), although the control group had a longer median CTx duration than the PJP group (123 days vs. 97 days). The types and frequency of chemotherapeutic agents showed no statistical difference between PJP group and control group. RTx or concurrent chemoradiation therapy (CCRTx) were more common in the PJP group, compared to the control group (RTx: 77.7% vs. 64.3%, *p* = 0.009; CCRTx: 39.3% vs. 21.7%, *p* < 0.001). Surgery was more common in the control group (11.6% vs. 23.8%, *p* = 0.006). The median daily steroid dose and proportion of steroid use were similar in the two groups (59.8% vs. 55.4%, *p* = 0.409). The median cumulative steroid dose tended to be higher in the PJP group (915 mg vs. 754 mg, *p* = 0.062) and the duration of steroid use was significantly longer in the PJP group (42 days vs. 26.5 days, *p* = 0.005). Patients with prolonged high-dose steroid use (20 mg of prednisolone equivalent per day for ≥3 weeks) were more common in the PJP group, compared to the control group (30 patients [26.8%] vs. 47 patients [14%], *p* = 0.002). The mean lymphocyte counts were 425/µL (range: 60–1,920/µL) in the PJP group and 540/µL (range: 10–2,730/µL) in the control group. The PJP group had a higher proportion of patients with lymphopenia (92% vs. 82.1%, *p* = 0.013). Only 30 of the 448 cases and controls (6.7%) received PJP prophylaxis, which was less frequent in the PJP group, compared to the control group (3 patients [2.7%] vs. 27 patients [8%], *p* = 0.049).

**Table 3:** Baseline characteristics of lung cancer patients according to PJP status.

Variable	PJP (n = 112)	Without PJP (n = 336)	*P*
Age (years), median (range)	69 (42–88)	69 (42–88)	0.995
Sex, male, n (%)	93 (83.0)	279 (83.0)	1.0
Histopathology, n (%)			0.286
Adenocarcinoma	52 (46.4)	166 (49.4)	
Squamous cell carcinoma	41 (36.6)	120 (35.7)	
Small cell lung cancer	14 (12.5)	48 (14.3)	
Stage, n (%)			0.586
I + II	8 (7.2)	32 (9.6)	
III	21 (18.7)	52 (15.5)	
IV	83 (74.1)	251 (74.9)	
Brain metastasis, n (%)	38 (33.9)	94 (28.0)	0.231
Surgery, n (%)	13 (11.6)	80 (23.8)	0.006
CTx, n (%)	106 (94.6)	311 (92.6)	0.452
Cisplatin	43 (40.6)	125 (40.3)	0.965
Carboplatin	64 (60.4)	168 (54.2)	0.268
Gemcitabine	23 (21.7)	69 (22.2)	0.917
Docetaxel	12 (11.3)	25 (8.1)	0.309
Paclitaxel	33 (31.1)	95 (30.6)	0.925
Pemetrexed	32 (30.2)	99 (31.9)	0.738
Etoposide	19 (17.9)	48 (15.5)	0.555
Molecularly targeted TKIs	29 (27.4)	107 (34.5)	0.189
Immune checkpoint inhibitors	10 (9.4)	17 (5.5)	0.157
CTx duration (days), median (range)	97 (0–1106)	123 (0–1393)	0.044*
RTx, n (%)	87 (77.7)	216 (64.3)	0.009
RTx cumulative dose (Gy), median (range)	54 (0–143)	60 (3–205)	0.023*
CCRTx, n (%)	44 (39.3)	73 (21.7)	<0.001
Steroid use, n (%)	67 (59.8)	186 (55.4)	0.409
Steroid ≥20 mg/d and ≥3 wks, n (%)	30 (26.8)	47 (14.0)	0.002
Steroid, daily dose (mg), median (range)^†^	25 (5–100)	25 (2–156.2)	0.476
Steroid, duration (days), median (range)^†^	42 (6–210)	26.5 (2–1363)	0.005
Steroid, cumulative dose (mg), median (range)^†^	915 (120–5250)	754 (30–6815)	0.062
Lymphopenia (<1000 cells/µL), n (%)	103 (92.0)	276 (82.1)	0.013
Lymphocyte count (cells/µL), median (range)	425 (60–1920)	540 (10–2730)	0.008*
PJP prophylaxis, n (%)	3 (2.7)	27 (8.0)	0.049

Data are presented as median (range) or numbers (percentages) unless otherwise indicated.

*Compared using Mann-Whitney *U* test.

^†^Data were obtained from 253 patients who ever used steroid during study period.

Steroid; prednisolone or equivalent dose.

PJP, *pneumocystis jirovecii* pneumonia; CTx, chemotherapy; TKI, tyrosine kinase inhibitor; RTx, radiotherapy; CCRTx, concurrent chemoradiotherapy.

### Risk factors for PJP

In the univariate analyses (Table 4), PJP development was significantly associated with RTx (OR: 1.95, 95% CI: 1.18–3.25; *p* = 0.001), CCRTx (OR: 2.59, 95% CI: 1.58–4.25; *p* < 0.001), lymphopenia (OR: 2.68, 95% CI: 1.23–5.82; *p* = 0.013), and prolonged high-dose steroid therapy (OR: 2.49, 95% CI: 1.42–4.37); *p* = 0.002). PJP development was inversely but non-significantly associated with TMP-SMX prophylaxis (OR: 0.33, 95% CI: 0.10–1.09; *p* = 0.068). In the multivariate analyses, PJP development was independently associated with prolonged high-dose steroid therapy (OR: 1.96, 95% CI: 1.06–3.63; *p* = 0.032) and CCRTx (OR: 2.09, 95% CI: 1.27–3.43; *p* = 0.004). Surgery also was inversely associated with PJP development in the multivariate analysis (OR: 0.42, 95% CI: 0.22–0.79; *p* = 0.008), although this is likely because patients who were able to undergo surgery would be less likely to receive CTx or RTx for their early-stage cancer.

**Table 4:** Clinical risk factors for PJP development.

Variable	Univariate		Multivariate	
	OR (95% CI)	*P*	OR (95% CI)	*P*
Brain metastasis	1.27 (0.80–2.02)	0.307		
Surgery	0.41 (0.22–0.73)	0.006	0.42 (0.22–0.79)	0.008
CTx	1.43 (0.57–3.60)	0.450		
RTx	1.95 (1.18–3.25)	0.001		
CCRTx	2.59 (1.58–4.25)	<0.001	2.09 (1.27–3.43)	0.004
Lymphopenia (<1000 cells/µL)	2.68 (1.23–5.82)	0.013		
Steroid ≥20 mg/d and ≥3 wks^†^	2.49 (1.42–4.37)	0.002	1.96 (1.06–3.63)	0.032
PJP prophylaxis	0.33 (0.10–1.09)	0.068		

^†^Prednisolone or equivalent dose.

PJP, *pneumocystis jirovecii* pneumonia; CTx, chemotherapy; RTx, radiotherapy; CCRTx, concurrent chemoradiotherapy.

## Discussion

Lung cancer is the most common cause of cancer death worldwide although the survival rate has improved due to the use of novel targeted therapies plus conventional therapies^24^. Disease progression itself may have influenced the prognosis and patient’s survival, infection and pneumonia are typically associated with poor prognoses among these immunocompromised patients^25^. Bacterial pneumonia is relatively common in this setting, compared to PJP, although recent studies have also indicated that PJP occurs among patients with solid malignancies, such as lung cancer^3,26–28^. Thus, the present study compared patients who had lung cancer with or without PJP, and the results revealed that prolonged high-dose steroid use and CCRTx were independent risk factors for developing PJP in this setting.

Long-term corticosteroid use is a known risk factor for developing PJP among non-HIV-infected patients^29,30^, which is likely related to their effects on T-cell immunity^8^. However, the present study detected a relatively small proportion of patients with steroid use and relatively low cumulative steroid dose in the PJP group, compared to the findings from previous studies^12,18^. This discrepancy may be related to differences in the definition of steroid use and other risk factors that might influence PJP development. For example, the present study excluded cases from the steroid use group if the steroid treatment was stopped at >1 month before the PJP diagnosis. In addition, we excluded cases with intermittent steroid use, and compared the maximum cumulative continuous steroid dose in the PJP and control groups. As a result, we did not detect a significant inter-group difference in daily steroid dose or cumulative maximum dose. However, when we categorized the patients according to daily steroid dose and duration, we found that a specific cut-off value (20 mg of prednisolone equivalent per day for ≥3 weeks) was independently associated with PJP development, although the area under the curve was low (0.568).

Previous studies have indicated that RTx could be a risk factor for PJP development^5,31^. In this study, univariate analysis revealed the RTx was associated with PJP development, this association was statistically insignificant in the multivariate analysis. Other reports have indicated that specific chemotherapeutic agents, such as fluorouracil, bleomycin, asparaginase, fludarabine, and gemcitabine, were associated with the risk of PJP development^3,27,28,32^. Patients who receive high-dose steroids with cytotoxic CTx or RTx could be even more vulnerable to PJP development^28^. In the current study, specific chemotherapeutic agents did not increase the risk of PJP, while CCRTx was significantly associated with PJP development in multivariate analysis (*p* = 0.004).

The present study revealed that most patients who developed PJP had decreased lymphocyte counts (median: 425/µL, range: 60–1,920/µL), which was a significant risk factor in the univariate analysis but not in the multivariate analysis. Mansharamani *et al*. have suggested that a cut-off value of 300 CD4+ cells/µL would include 91% of PJP cases among HIV-negative patients, which is a high-risk group, although 39–46% of individuals who received long-term corticosteroid therapy also had CD4+ counts of <300/µL^33^. Moreover, the accuracy of this biomarker is poorly characterized for non-HIV-infected patients, which limits its use in that population, although it is an accurate method for assessing PJP risk among patients with AIDS^18,34^.

The characteristics of our patients with PJP and lung cancer were different from those of non-HIV-infected patients in other studies. The most common clinical symptom was dyspnea in the present study, and our patients’ respiratory symptoms tended to manifest quickly, which was also observed in previous studies^12,35^. However, the frequencies of fever and cough were relatively low in our study, compared to previous studies^12,35^. This discrepancy may be related to reduced immunity caused by CTx and/or RTx, which could mask signs of infection. The reported prognosis of PJP is poorer among non-HIV-infected patients, compared to HIV-infected patients^36^, although we observed a much higher mortality rate during PJP treatment (approximately 60%), compared to other studies conducted in non-HIV-infected patients^5,35^. In this current study, most patients received first-line TMP-SMX treatment and approximately 67% of the patients with PJP also received high-dose adjunctive steroids during their PJP treatment^19^. Thus, the poor prognosis may be related to the advanced lung cancer and/or the weakened general condition of patients who receive CTx and/or RTx before developing PJP. It is also possible that the large proportion of patients with do-not-resuscitate or do-not-intubate statuses was responsible for the relatively high mortality rate among our patients with lung cancer and PJP.

Among HIV-infected patients, TMP-SMX prophylaxis is highly effective for preventing PJP^34^. A recent meta-analysis also indicated that, among solid organ transplant recipients and patients with hematological malignancies, TMP-SMX treatment was associated with reductions in the occurrence of PJP (RR: 0.09, 95% CI: 0.02–0.32) and PJP-related mortality (RR: 0.17, 95% CI: 0.03–0.94), which suggests that PJP prophylaxis is warranted when adult patients have a >3.5% risk of PJP^11^. Several studies have indicated that solid malignancies are associated with an increased risk of PJP^3,12,28,37^, although there are no clinical data supporting the routine use of PJP prophylaxis for patients with solid tumors. The American Thoracic Society recommends considering prophylaxis when immunocompromised patients (including patients with solid tumors) receive prolonged high-dose corticosteroid treatment (20 mg of prednisolone equivalent per day for ≥4 weeks), although it acknowledges that this recommendation is based on a low level of evidence^38^. The present study also confirmed that PJP prophylaxis is not routine for patients with lung cancer, even for patients who are receiving prolonged high-dose steroid therapy. As PJP prophylaxis can significantly reduce mortality among patients with other diseases, it may be beneficial to provide PJP prophylaxis to high-risk patients with lung cancer. For example, prolonged high-dose steroid therapy and CCRTx were possible risk factors for PJP among patients with lung cancer, and the most common reason for steroid use was radiation-induced pneumonitis, which is relatively common after CCRTx (vs. RTx)^21^. Therefore, it seems reasonable to use PJP prophylaxis for patients who are receiving prolonged high-dose steroid treatment and CCRTx.

The present study has several limitations. First, the single-center retrospective design is associated with known risks of bias, and we were unable to collect exact information regarding symptom presentation or duration before the PJP diagnosis. Thus, our findings may not be generalized to other centers or patient populations. Second, proportion of surgery in PJP cases and controls are somewhat different, suggesting the possibility of inadequate cancer stage matching. Third, we used PCR and immunofluorescence data to diagnose PJP, and our data may include false-positive results, as a confirmed PJP diagnosis generally requires special microbiological staining of respiratory specimens^39^. However, non-HIV-infected patients have a lower overall organism burden^40^ compared to HIV-infected patients, and conventional staining methods might have provided lower sensitivity and false-negative results. A meta-analysis of PCR-based PJP diagnosis revealed a sensitivity of 99% and specificity of 92% among non-HIV-infected patients, and that 31.8% of patients with false-positive results had or subsequently developed PJP^41^. Thus, patients with a positive PCR result require careful follow-up and/or PJP treatment. Other reports have also suggested that PCR assays for PJP are sensitive and increase the diagnostic yield among non-HIV-infected immunocompromised patients^16,42,43^. Therefore, to reduce the likelihood of false-positive results, we only included patients with symptoms of acute pulmonary infiltrates, such as cough, dyspnea, and radiological abnormalities.

Despite its limitations, the present study also has several strengths. To the best of our knowledge, this is the first case-control study of PJP among patients with lung cancer. In addition, we matched the patients with PJP to a relatively large number of control patients, which provides a clearer perspective regarding the risk factors for PJP development among patients with lung cancer. Furthermore, our findings may be useful for identifying high-risk patients with lung cancer who should receive PJP prophylaxis. Nevertheless, multicenter randomized controlled trials are needed to examine the value of PJP prophylaxis in this setting.

## Conclusion

In conclusion, the present study revealed that prolonged high-dose steroid therapy (20 mg of prednisolone equivalent per day for ≥3 weeks) and CCRTx were risk factors for PJP development among patients with lung cancer. These patients had a very poor prognosis. Furthermore, the most common reasons for the steroid treatment were CCRTx or RTx-induced pneumonitis. Therefore, clinicians should consider PJP prophylaxis for high-risk patients with lung cancer.

## Data Availability

All datasets generated and analysed during this study are available from the corresponding author on reasonable request.
